# Mental Imagery, Impact, and Affect: A Mediation Model for Charitable Giving

**DOI:** 10.1371/journal.pone.0148274

**Published:** 2016-02-09

**Authors:** Stephan Dickert, Janet Kleber, Daniel Västfjäll, Paul Slovic

**Affiliations:** 1 Department of Marketing, WU Vienna University of Economics and Business, Vienna, Austria; 2 Department of Psychology, Linköping University, Linköping, Sweden; 3 Department of Psychology, Alpen-Adria University of Klagenfurt, Klagenfurt, Austria; 4 Decision Research, Eugene, Oregon, United States of America; 5 Department of Psychology, University of Oregon, Eugene, Oregon, United States of America; The University of Chicago, UNITED STATES

## Abstract

One of the puzzling phenomena in philanthropy is that people can show strong compassion for identified individual victims but remain unmoved by catastrophes that affect large numbers of victims. Two prominent findings in research on charitable giving reflect this idiosyncrasy: The (1) identified victim and (2) victim number effects. The first of these suggests that identifying victims increases donations and the second refers to the finding that people’s willingness to donate often decreases as the number of victims increases. While these effects have been documented in the literature, their underlying psychological processes need further study. We propose a model in which identified victim and victim number effects operate through different cognitive and affective mechanisms. In two experiments we present empirical evidence for such a model and show that different affective motivations (donor-focused vs. victim-focused feelings) are related to the cognitive processes of impact judgments and mental imagery. Moreover, we argue that different mediation pathways exist for identifiability and victim number effects.

## Introduction

Humanitarian aid crises occur regularly throughout the world, including ongoing threats to human life from natural and man-made disasters. In response to these catastrophes, a substantial amount of money is donated annually to alleviate the suffering of those affected. A large portion of these donations (i.e., 72% of the $358 billion given in the US in 2014) comes from individual donors [1]. However, exactly how and when people decide to donate is still a hotly debated topic. Research suggests that these types of decisions are multi-faceted and often determined by many factors [2].

One of the central phenomena in research on philanthropy is the finding that people experience much empathy for individual victims but can remain largely unmoved by catastrophes that affect many people [3, 4]. Moreover, as the number of people at risk increases, helping responses can become scope insensitive (i.e., they are not sensitive to the number of people in need; [5]). Several studies have implicated different affective and cognitive mechanisms essential in the perception and processing of information related to valuations of lives (e.g., [6–20]).

Two prominent findings highlight the importance of understanding the psychological processes underlying the valuation of lives in charitable contexts: The (1) identified victim and (2) victim number effects. The former refers to the fact that identifying the people in need often increases donors’ willingness to contribute to a charitable cause (e.g., [8]). The latter refers to the negative effect of being confronted with larger numbers of victims on donors’ willingness to help (e.g., [12]).

### Processes underneath identifiability and victim number effects

Both identifiability and victim number effects have been linked to affective processes. Affective explanations are based on the idea that helping behavior is often motivated by pro-social emotions [21]. Presenting identified (vs. unidentified) victims is assumed to result in higher donations because they generate stronger emotions in the donor [12, 13, 17, 22]. It is easier to attach emotional impressions to an identified victim due to a more concrete mental representation [3, 23, 24]. Presenting an unidentified victim, on the other hand, may engage less affective processing and reduce people’s willingness to help [18, 25]. The effect of identification is not limited to pro-social emotions and charitable giving [10, 11, 26] and can also decrease helping when victims can be blamed for their predicament [11]. However, even in these circumstances the effect of identification can be explained by stronger emotional reactions. Evidence exists that the positive effect of identification on donations is particularly strong for single victims [12]. Charities often make use of this fact by presenting single, individual victims to represent a specific humanitarian aid cause. Instead of showing the staggering statistics of people in need, presenting a single individual is a common practice to increase donations.

The singularity effect represents a specific form of insensitivity to the number of victims. As the number of lives at risk increases, donors can become less inclined to contribute in general [3]. Moreover, being confronted with many lives at risk can have a detrimental effect on donors’ willingness to contribute even to a single victim. Supporting this notion, Small and colleagues [18] found that presenting background statistics about the total number of lives at risk reduced participants’ donation amounts to one victim. Their results can be interpreted as evidence for an affective account of the “drop-in-the-bucket effect”, such that the inclusion of background statistics hinders the generation of prosocial emotions.

However, recently an alternative explanation has been proposed that capitalizes on the importance of perceived impact of a donation [27–31]. Presenting background statistics can make it obvious that helping only a few victims will not alleviate the suffering of all (or even most) people affected and therefore each individual donation does not seem to make much of a difference in the big picture. Evaluating the impact (i.e., usefulness) of a donation against the backdrop of a much larger tragedy can lead to drop-in-the-bucket effects and demotivate giving. Research on perceived impact suggests that affective responses (such as empathy) play a smaller role in donation decisions than previously assumed. For example, Erlandsson and colleagues [29]; see also [30] found that the perceived utility of a donation mediates the proportion dominance effect in charitable giving (i.e., people preferring to help a larger proportion even if this means helping a lower absolute number of victims). Likewise, Cryder and colleagues [27] suggest that the perceived impact of a donation may be a better predictor than sympathy towards charitable causes.

### The dance of affect and impact

It is unclear to which extent explanations based on impact judgments can supplant affective accounts of identifiability and victim number effects. Considering impact judgments as a motivator for charitable giving has intuitive merit because donors like their donation to be effective. It may also be difficult to rationalize spending money on something that has little chance of succeeding. While this line of reasoning does not dispute that identifiability and victim number effects can operate through more concrete mental images, the effects of these images are not assumed to be affective in nature [27]. However, it is also possible that impact judgments give rise to emotions instrumental in prosocial behavior.

While feelings related to the victims (e.g., sympathy and compassion) might not depend on impact judgments, feelings related to the donor (e.g., feeling good about oneself for donating or regretting not supporting a charitable cause) certainly could. If a particular project is deemed to be effective, donors may feel good about themselves for supporting it. This feeling has been described as “warm glow” in the literature [32, 33] and in our work we have also found donor-focused feelings to correspond to impact judgments and donations [25]. Just like impact judgments, donor-focused feelings could be reduced when the donation is perceived as a drop in the bucket (e.g., when victim statistics are presented). To get a better understanding of the effects of impact judgments and how they influence donor- and victim-focused feelings, a more thorough investigation is needed.

### Overview of studies

Here we propose a model that incorporates both explanations (i.e., perceived impact and affect) and highlights how the different processes are related to each other as well as to donations. We hypothesize that the processes through which identifiability influences donations are primarily related to mental imagery and victim-focused feelings (e.g., sympathy). This reasoning is in line with Slovic [3], who argued that clearer mental images give rise to empathic feelings (see also [24] for a more general argument on mental imagery and affect). In the current studies mental imagery refers to the coherence and cohesiveness by which an image of the victim(s) can be mentally formed. Thus, we conceptualize mental imagery by the consistency and unity of a mental representation.

Conversely, the effect of changes in victim number may be more related to the perceived impact of a donation [29] and subsequent feelings related to the donor (i.e., to regret not donating). As the number of lives at risk increases, it is likely that any donation is seen as less effective and people will be less likely to regret withholding their financial support [34]. We postulate that although impact judgments are an important consideration, their effect on donation decisions is best understood by their influence on affective reactions of the donor. Based on this reasoning, we expect the following mediation:

1. Identifiability → Mental Image → Victim-focused emotions (Sympathy) → Donations
2. Victim Number → Impact → Donor-focused emotions (Regret) → Donations

To test these pathways, we present two studies that manipulate identifiability and number of lives at risk. Whereas Study 1 is a reanalysis of an already published data set on the effects of victim numbers and identifiability on hypothetical donation amounts [6], Study 2 is a conceptual replication that uses real donations and manipulates victim numbers through the presentation of background statistics.

### Ethics statement

All studies in this paper were conducted in accordance with all applicable human subjects guidelines, procedures, rules, and regulations in effect in the country in which the research was conducted, and in full accordance with international ethics for research and human subjects guidelines, including the 1964 Declaration of Helsinki.

Study 1 was conducted at the Max Planck Institute for Research on Collective Goods, Bonn, Germany. The Max Planck Institute does not have an IRB from which to obtain ethical approval. No formal body at the Institute is empowered to grant approval for research not of a clinical or medical nature (and Study 1 does not constitute either clinical or medical research), nor does German law require such approval. Therefore the work conducted in Germany complied with German law and followed ethical guidelines for research at the Max Planck Institute. Study 2 was conducted in Eugene, Oregon, USA and was approved by the local IRB at the University of Oregon.

In both studies, participants received information about the study and gave their written informed consent prior to participating, they were compensated for their participation, procedures were in place to protect their confidentiality, and they were thoroughly debriefed.

Data for Study 1 and Study 2 can be found in S1 Dataset 1 and S2 Dataset 2.

## Study 1: Hypothetical Donation Request

### Method

#### Participants

One-hundred and sixty-eight participants (*M* = 23.9, *SD* = 5.1; 62% women) took part in Study 1, which was included in a larger experimental battery that lasted about one hour. Participants were recruited from a subject pool of the University of Bonn, Germany, and included students as well as community members. They were compensated with 12 Euros (approximately $15) for their participation.

#### Design, Materials, and Procedure

As part of this 2 (identified vs. unidentified) x 2 (number of victims: 1 vs. 5) between-subjects study, participants were first presented with hypothetical donation requests that depicted either one or five African children. Participants were told that these children are in danger of starvation and that donations to an internationally accredited humanitarian aid organization working with these children will help reduce their hunger. Children were either identified by pictures and names (identified condition) or remained nameless and faceless (unidentified condition). In the identified condition, participants either saw one picture or five pictures, whereas in the unidentified condition the number of children in need of help was communicated by a number (1 vs. 5). Participants were further told that this child was (these five children were) part of a larger group of 100 children who faced the same predicament and also needed financial help to survive. However, participants were only able to contribute to the one child (or five children). After reading the donation request, participants indicated their donation amount and (on a scale of 1 = “not at all” to 7 = “very much”) how much sympathy they felt towards the one child (five children), how much they would regret not donating, how coherent and cohesive their mental impression of the child (five children) was, and how much a donation would improve (i.e., impact) the life of the child (five children).

### Results

#### Data analyses

In this part of the analysis we were primarily interested in the mediating role of impact judgments, mental images, as well as donor and victim-focused feelings (i.e., regret and sympathy, respectively). We will first present a mediation analysis of the hypothesized pathways with bootstrapping according to Preacher and Hayes [35] and then use structural equation modeling (SEM) to conduct a path analyses and further evaluate model fit and alternative mediation models. Prior to analyses, donation amounts were winsorized at 250 Euros (less than 6% of the data) and log-transformed (ln(x+1)) to reduce skewness (for descriptive data of all variables, see Table 1). For the mediation analyses of identifiability effects, the data were collapsed across victim numbers. Similarly, for the mediation analyses for victim number effects, the data were collapsed across identifiability.

**Table 1:** Descriptive Statistics for Study 1 and Study 2.

	Study 1					Study 2				
	All	Ident Single	Ident Group	Unident Single	Unident Group	All	Ident Single	Ident Statistics	Unident Single	Unident Statistics
Willingness to donate	80.4%	88.1%	85.0%	67.4%	81.4%	67.3%	69.0%	69.0%	69.0%	61.9%
Donations (raw)	45.96 (67.27)	64.05 (77.22)	35.18 (48.59)	33.16 (55.91)	51.14 (78.87)	2.84 (3.67)	2.76 (4.02)	2.71 (2.98)	3.26 (4.23)	2.62 (3.42)
Donations (transform)	2.70 (1.74)	3.22 (1.69)	2.72 (1.52)	2.15 (1.84)	2.74 (1.78)	0.98 (0.84)	0.94 (0.84)	1.01 (0.80)	1.04 (0.90)	0.93 (0.85)
Mental Image	3.34 (1.72)	3.81 (1.67)	3.60 (1.75)	3.02 (1.75)	2.95 (1.59)	3.04 (1.74)	3.60 (1.87)	3.12 (1.63)	2.86 (1.75)	2.62 (1.64)
Sympathy	5.31 (1.50)	5.60 (1.33)	5.13 (1.44)	5.28 (1.44)	5.24 (1.76)	5.10 (1.59)	5.39 (1.34)	5.33 (1.44)	4.64 (1.67)	5.02 (1.80)
Impact	4.02 (1.70)	4.10 (1.79)	3.83 (1.28)	4.02 (1.87)	4.14 (1.79)	2.82 (1.57)	3.12 (1.74)	2.69 (1.42)	2.95 (1.65)	2.50 (1.41)
Regret	3.66 (1.88)	3.76 (1.88)	3.60 (1.66)	3.60 (1.95)	3.67 (2.06)	3.67 (2.12)	3.78 (2.20)	3.54 (2.20)	4.02 (1.89)	3.36 (2.20)
N	168	42	40	43	43	168	42	42	42	42

*Note*. Donations (transform) refer to winzorised and log-transformed donation amounts in Study 1 and log-transformed donation amounts in Study 2; Donations (raw) refer to winzorised donation amounts in Study 1 (at 250 Euro) and raw donations in Study 2. Ident = Identified Victim Condition, Unident = Unidentified Victim Condition; Means are presented with their standard deviation in parentheses.

#### Mediation of identifiability effects

To test for possible mediation of identifiability effects through mental images and sympathy, we conducted a set of analyses with multiple mediators ([35, 36]). Results are presented in Table 2. As expected, identifiability predicted mental images, (*M*_Identified_ = 3.71, *SD* = 1.70; *M*_Unidentified_ = 2.99, *SD* = 1.66), ß = .20, *p* = .008. Moreover, more coherent mental images predicted higher sympathy, ß = .17, *p* = .029, and higher sympathy predicted greater donation amounts, ß = .29, *p* < .001. Importantly, a significant indirect effect emerged from the data, *b* = .010, 95%-CI [.001, .032]. Thus, the effect of identifiability on donation amounts can be explained by the serial mediating effect of mental imagery and sympathy. Once this indirect effect on donations is accounted for, neither identifiability nor mental images significantly predicted donations. These results suggest that people donate more to identified victims by virtue of the mental images these victims induce. More coherent mental images, in turn, can lead to stronger sympathy for the donation recipients. Further analyses also revealed that the indirect effect of identifiability on donations cannot be explained by impact judgments and regret as serial mediators, *b* = -.005, 95%-CI [–.027, .017].

**Table 2:** Summary of the Main Mediation Results with Bootstrapping for Study 1 and Study 2.

	Study1	Study 2
Analyses for Identifiability Effects		
*Direct effects*		
Identifiability → Image	ß = .20^**^	ß = .18^*^
Image → Sympathy	ß = .17^*^	ß = .37^***^
Identifiability → Sympathy	ß < .01	ß = .10
Image → Donation	ß = .10	**ß = .14**^**+**^
Sympathy → Donation	ß = .29^***^	ß = .08
Identifiability → Donation	ß = .12	ß = –.06
*Indirect effects*		
Identifiability → Image → Sympathy → Donation	**CI [.001, .032]**, *b* = .010; *R*^*2*^ = .129	CI [–.003, .027], *b* = .005; *R*^*2*^ = .040
Identifiability → Sympathy → Image → Donation	CI [–.001, .011], *b* = .001; *R*^*2*^ = .129	CI [–.001, .030], *b* = .010; *R*^*2*^ = .040
Identifiability → Impact → Regret → Donation	CI [–.027, .017], *b* = -.005; *R*^*2*^ = .400	CI [–.012, .034], *b* = .008; *R*^*2*^ = .224
*N*	167	166
Analysis for Victim Number Effects		
*Direct effects*		
Victim number → Impact	ß = –.02	**ß = –.14**^**+**^
Impact → Regret	ß = .32^***^	ß = .30^***^
Victim number → Regret	ß = –.01	ß = –.04
Impact → Donation	ß = .34^***^	ß = .17*
Regret → Donation	ß = .41^***^	ß = .40^***^
Victim number → Donation	ß = .03	ß = .03
*Indirect effects*		
Victim number → Impact → Regret → Donation	CI [–.023, .018], *b* = -.003; *R*^*2*^ = .375	**CI [–.055,–.001]**, *b* = -.017; *R*^*2*^ = .224
Impact → Regret → Donation	**CI [.066, .203]**, *b* = .129; *R*^*2*^ = .374	**CI [.056, .235]**, *b* = .123; *R*^*2*^ = .223
Victim number → Regret → Impact → Donation	CI [–.020, .017], *b* = -.001; *R*^*2*^ = .375	CI [–.025, .001], *b* = -.004; *R*^*2*^ = .224
Victim number → Image → Sympathy→Donation	CI [–.015, .005], *b* = -.002; *R*^*2*^ = .118	CI [–.022, .002], *b* = -.003; *R*^*2*^ = .039
*N*	168	159

*Note*. Parameters are estimated for full models; varying N results from missing values; indirect effects were estimated at point estimates and 95% confidence intervals with 1000 bootstrapping samples; significant CIs do not include zero and are in boldface; Image = Mental Image

^+^*p* ≤ .10

^*^p ≤ .05

^**^p ≤ .01

^***^p ≤ .001

Analyses with non-transformed data revealed similar patterns of results.

#### Mediation of victim number effects

We next analyzed whether manipulations of victim number operate indirectly through impact judgments and regret. Contrary to our expectations, we did not find any direct (*p* > .667) or indirect effects, *b* = -.003, 95%-CI [–.023, .018] of victim numbers on donations through these mediators. However, independently of the victim number manipulation we found a direct effect of impact judgments on donations, ß = .34, *p* < .001, a direct effect of regret on donations, ß = .41, *p* < .001, and a significant indirect effect of impact judgments through regret on donations, *b* = .129, 95%-CI [.066, .203]. This result suggests that the effect of impact judgments on donations, at least partially, can be explained by its influence on participants’ regret towards not donating. Further analyses also showed that no indirect effect emerged when using mental imagery and sympathy as serial mediators for victim number, *b* = -.002, 95% CI [–.015, .005].

#### Path analysis: Identifiability

We used Stata 13 [37] to conduct a path analysis with SEM in order to further test the hypothesized mediation and evaluate the likelihood of competing models that include the non-hypothesized pathways and the reversed order of mediators. Consistent with the analyses reported above, we examine the effects of identifiability and victim number separately. In this analysis we consider models with one and two pathways from the independent variable to donations, each pathway consisting of two mediators. Direct effects of identifiability and victim number on donations are not included, as we are primarily interested in the indirect effects. However, we do allow direct effects of all mediators to the dependent variable.

The hypothesized indirect pathway for the effect of identifiability on donation amounts with mental images and sympathy as serial mediators (Model 1, see Table 3) showed good model fit (i.e., this model was not significantly different from a fully specified model, Χ^2^ (2, *N* = 168) = 2.7, *p* = .26) with a CFI = .977 and RMSEA = .046 (usually values of CFI > .90 and RMSEA < .05 provide evidence for good model fit [38]). Reversing the order of mediators (i.e., sympathy → mental images; Model 2) reduces model fit (CFI = .732 and RMSEA = .155) and produces a model significantly worse than a fully specified model, X^2^ (2, *N* = 168) = 10.04, *p* < .01. To test which order of mediators better fits the data, we use Bayesian information Criteria (BIC). Smaller BICs indicate better model fit, and the difference in BICs can be used to compare models. We will follow Raftery’s [39] recommendation of interpreting differences in BICs of two models in the following way: BIC differences of 0–2 are interpreted as weak evidence, BIC differences of 2–6 are indicative of positive evidence, BIC differences of 6–10 are indicative of strong evidence, and BIC differences > 10 are indicative of very strong evidence for the superior model (see also [40]). Thus, the observed difference in BICs (ΔBIC = 7.4) for the two models (Models 1 & 2) provides strong evidence for the hypothesized order of mediators (i.e., mental images → sympathy), with a posterior probability for the hypothesized model Pr(Model 1|ΔBIC = 7.4) = .976.

**Table 3:** Model Fit for Path Analyses, Study 1.

Model	Pathway	X^2^	Model parameters	CFI	RMSEA	BIC
**1**	ID—MI—SY—DO	2.7	4	.977	.046	2421.5
**2**	ID—SY—MI—DO	10.04**	4	.732	.155	2428.9
**3**	ID—MI—SY—DO & ID—IM—RE—DO	37.56***	8	.776	.161	3723.6
**4**	ID—MI—SY—DO & IM—RE—DO	37.77***	7	.781	.149	3718.7
**5**	VN—IM—RE—DO	0.19	4	1	< .01	2435.1
**6**	VN—RE—IM—DO	0.25	4	1	< .01	2435.1
**7**	VN—IM—RE -DO & VN—MI—SY-DO	33.16***	8	.790	.149	3731.1
**8**	VN—IM—RE—DO & MI—SY—DO	33.47***	7	.795	.138	3726.3

*Note*. X^2^ denotes model fit compared to a fully specified model with all pathways specified. Model parameters represent number of estimated pathway coefficients. ID = identifiability; MI = mental imagery, SY = sympathy; VN = victim number; IM = impact judgments; RE = regret; DO = donations.

**p < .01

***p < .001

We next examined whether adding the indirect pathway through impact and regret improved model fit (Model 3). Comparing the model that specifies two indirect pathways against the hypothesized model with only indirect effects through mental imagery and sympathy shows very strong support for the hypothesized model, Pr(Model 1|ΔBIC = 1302.1) > .999. We also compared Model 3 to a reduced model in which all four mediators are present but the pathway from identifiability to impact was constrained to 0 (Model 4). A likelihood ratio test revealed that including the indirect pathway to impact judgments (and therefore the indirect pathway through the non-hypothesized mediators; Model 3) does not improve model fit compared to Model 4, X^2^(1, *N* = 168) = 0.2, *p* = .651. In fact, comparing the differences in BICs (ΔBIC = 4.9) provides evidence that Model 4 is more likely than Model 3, Pr(Model 4|ΔBIC = 4.9) = .92. These analyses provide strong support for the hypothesis that the effects of identifiability are mediated by mental imagery and sympathy (in this order) in Study 1.

#### Path analysis: Victim number

A similar path analysis was conducted to examine the model fit for the effects of victim number on donations with impact and regret as serial mediators. Results showed that the models with a single pathway (Models 5 & 6, Table 3) fit the data equally well and are not significantly different from a fully specified model, X^2^ (2, *N* = 168) < 0.25, *p*s > .88. We find no evidence that one order of mediators is superior (ΔBIC = 0.6). This is likely the result of allowing each mediator to directly correlate with donations. In fact, when constraining the models to only include indirect effects (and no direct pathway from the first mediator to donations) then the model with the hypothesized order of mediators (Model 5) outperforms the competing model (Model 6), Pr(Model 5| ΔBIC = 9.7) = .993, and suggests that impact judgments precede participants’ regret. Including the other indirect pathway (mental image → sympathy; Model 7) did not improve model fit in comparison to Model 8, which constrains the pathway from victim numbers to mental image to 0, X^2^ (1, N = 168) = 0.31, *p* = .578. Moreover, given the differences in BICs, Model 7 was less likely than Model 8, Pr(Model 8|ΔBIC = 4.8) > .917, and the hypothesized Model 5, Pr(Model 5|ΔBIC = 1296) > .999. However, contrary to our expectations and as already revealed by the mediation analysis with bootstrapping, the indirect pathway of victim number on donations through impact and regret was not significant, *z* = –0.27, *p* = .786.

### Discussion

Taken together, results from both the mediation analyses with bootstrapping and path analyses suggest that the effects of identifiability on donations can best be explained through their influence on mental imagery and, subsequently, sympathetic responses. This is in line with prior research highlighting the specific cognitive underpinnings of affective responses in prosocial behavior [3, 6, 27].

Contrary to our expectations, no significant indirect effects of increasing victim number on donations through impact and regret emerged from the data. However, a separate mediation analysis found that, regardless of the number of victims, the effect of perceived impact was mediated by regret. One possibility for the lack of direct and indirect effects regarding the number of victims could be related to the fact that we did not use real donations in Study 1. Moreover, in Study 1 the manipulation of victim number included a constant reference group (i.e., 100) in both victim number conditions. This could have attracted attention away from the actual donation recipients (1. vs. 5) and masked differences in participants’ impact judgments, regret, and donation responses. It is also possible that impact judgments depend more on the total number of people at risk rather than the subset of people one can help.

## Study 2: Real Donation Request

To extend the findings from Study 1, we present a second study in which participants were confronted with real donation requests. We also examined the proposed mediation model when using a different manipulation of victim numbers. In Study 2, we increased the number of lives at risk by a background statistics manipulation similar to Small and colleagues [18]. In their design, participants were either provided with background statistics about the humanitarian aid problem (i.e., the total number of people in need of help) or received no information on background statistics. The identifiability manipulation in Study 2 was similar to the one used in Study 1 (name and picture vs. none).

### Method

#### Participants

For this study, 168 university students (undergraduate and graduate) and community members were recruited on campus at the University of Oregon (*M*_age_ = 25.8 years, *SD* = 7.6; 51% women). The study was part of a larger survey with unrelated experiments which lasted about one hour. As compensation for their efforts, participants received $14. In this Study, we collected real donations and participants could use their monetary compensation to donate to a child in need.

#### Design and Materials

This study employed a 2 (identified vs. unidentified) x 2 (background statistics vs. no background statistics) between-subjects design. Participants’ willingness to donate was assessed by asking for financial contributions to the humanitarian aid organization Save the Children to alleviate the severe food crisis in Southern Africa. Victim identifiability was manipulated by presenting half of the participants with identifying information of the victim while the other half was only informed that they would be able to donate to “a child in Africa.” In the identified condition, participants were presented with a picture, the name (Rokia) and age (7 years old) of the child in need. In the unidentified condition, participants only read about a child in need and were not given any identifying information. Specifically, the story about the child was presented as follows:

“Rokia (the child) is desperately poor, and faces a threat of severe hunger or even starvation. Her (his or her) life will be changed for the better as a result of your financial gift. With your support, and the support of other caring sponsors, Save the Children will work with Rokia’s (this child’s) family and other members of the community to help feed her and provide her (him or her) with education, as well as basic medical care and hygiene.”

Victim numbers were conceptualized as the presence versus absence of background statistics on the total number of children at risk. Specifically, half of the participants were told that Rokia (the child) is “one of 20,000 needy children in the same region who are desperately poor.” The other half of the participants were not shown any background statistics and were only informed about Rokia (the one child).

#### Procedure

At the beginning of the experiment, participants were randomly assigned to one of the four conditions and given the study materials together with their participant fee ($14). They could donate any portion of this participant fee. Participants completed the materials individually and in a separate room. After reading the donation request, they were asked to indicate their donation amounts to Rokia (the child in need). Furthermore, we assessed participants’ feelings (e.g., sympathy and regret), mental image of the victim, and impact of a donation on 7-point response scales as in Study 1. Finally, to minimize experimenter demand effects on participants’ donations, participants were asked to put the completed questionnaire and their donation amount in an envelope, seal it and return it to the experimenter.

### Results and Discussion

#### Data analyses

Means and standard deviations for all dependent variables are presented in Table 1. We again used mediation analyses to look at both direct and indirect effects. Donation amounts were log-transformed prior to analysis. As in Study 1, we first present the results of a mediation analysis with bootstrapping and then conduct a path analyses with SEM.

#### Mediation of identifiability effects

A significant effect of identifiability on mental images demonstrated that images for the identified victim were more coherent (*M*_Identified_ = 3.35, *SD* = 1.75) than images for the unidentified victim (*M*_Unidentified_ = 2.74, *SD* = 1.69), ß = .18, *p* = .022. Likewise, more coherent mental images predicted higher sympathy, ß = .37, *p* < .001. Donation amounts are marginally predicted by mental image, ß = .16, *p* = .054, while donation amounts are not predicted by sympathy (ß = .08, *p* = .360) or identifiability (ß = –.05, *p* = .567). However, the predicted indirect effect with mental image and sympathy as mediators between identifiability and donation amounts did not reach conventional significance, *b* = .005, 95%-CI [–.003, .027]. A mediation with the reversed order of mediators, with sympathy preceding mental images, also did not show a significant effect, *b* = .010, 95%-CI [-.001, .030]. As in Study 1, a mediation of identifiability through impact judgments and regret did not find significant effects in Study 2, *b* = .008, 95%-CI [–.012, .034].

#### Mediation of victim number effects

While the direct effect of victim number on donation amounts was not significant, ß < .03, *p* = .646, we found a marginally significant direct effect of the victim number manipulation on impact judgments, ß = –.14, *p* = .081. Presenting a single victim resulted in slightly higher impact ratings (*M* = 3.04, *SD* = 1.68) than presenting a single victim with background statistics (*M* = 2.59, *SD* = 1.41). In the next step, regret is predicted by impact, ß = .30, *p* < .001, but not by victim number, ß = –.04, *p* = .569. Donation amounts are predicted by impact, ß = .17, *p* = .030 and regret, ß = .40, *p* < .001, as expected. Confirming our hypothesis regarding the mechanisms behind victim number effects, we found a significant indirect effect through impact and regret on donations, *b* = -.017, 95%-CI [–.055,–.001]. Reversing the order of mediators (i.e., first regret then impact judgments) did not reveal a significant indirect effect, *b* = -.004, 95%-CI [–.025, .001]. Accordingly, presenting victim statistics (and thereby increasing the total number of people in need) led to lower impact judgments, which predicted participants’ regret for not donating and subsequent donations. As in Study 1, we found no evidence for an indirect effect through mental imagery and sympathy for the manipulation of victim number, *b* = -.003, 95% CI [–.022, .002].

#### Path analysis: Identifiability

Next we present the path analyses for Study 2, which were conducted to evaluate model fit of the hypothesized and competing models. Results are presented in Table 4 and include separate analyses for identifiability and victim number effects.

**Table 4:** Model Fit for Path Analyses, Study 2.

Model	Pathway	X^2^	Model parameters	CFI	RMSEA	BIC
**1**	ID—MI—SY—DO	2.33	4	.979	.046	2158.5
**2**	ID—SY—MI—DO	2.91	4	.974	.052	2159.1
**3**	ID—MI—SY—DO & ID—IM—RE—DO	57.79***	8	.615	.208	3496.5
**4**	ID—MI—SY—DO & IM—RE—DO	58.39***	7	.618	.194	3491.9
**5**	VN—IM—RE—DO	1.39	4	1	< .01	2203.1
**6**	VN—RE—IM—DO	2.86	4	.984	.05	2204.6.1
**7**	VN—IM—RE -DO & VN—MI—SY-DO	57.92***	8	.612	.208	3497.3
**8**	VN—IM—RE—DO & MI—SY—DO	59.61***	7	.606	.196	3493.8

Note: X^2^ denotes model fit compared to a fully specified model with all pathways specified. Model parameters represent number of estimated pathway coefficients. ID = identifiability; MI = mental imagery, SY = sympathy; VN = victim number; IM = impact judgments; RE = regret; DO = donations.

***p < .001.

As in Study 1, we conducted a path analysis with SEM to examine the quality of different mediation models to explain identifiability effects. Results of Study 2 again show good fit for the model with the hypothesized indirect pathway with mental images and sympathy as serial mediators (Model 1), X^2^ (2, *N* = 168) = 2.33, *p* = .311) with a CFI = .979 and RMSEA = .046. Model fit remains good when reversing the order of mediators (Model 2), X^2^ (2, *N* = 168) = 2.91, *p* = .233, CFI = .974, RMSEA = .052. The difference in BICs for both models is relatively small, with no clear evidence for a particular order of mediators, Pr(Model 1|ΔBIC = 0.58) = .571. While the hypothesized indirect effect for Model 1 was marginally significant (z = 1.67, *p* = .094), the indirect effect for the reversed order of mediators was not (z = 1.35, *p* = .176). Using non-transformed data reveals a similar pattern of results, however the indirect effects do not reach conventional significance levels (*p* = .119 and *p* = .192 for Model 1 and 2, respectively.)

Next we tested whether adding the indirect pathway through impact and regret increased model fit (Model 3). As in Study 1, results show strong support for the model with only the hypothesized indirect pathway through mental images and sympathy, Pr(Model 1|ΔBIC = 1337.9) > .999. Comparing Model 3 to a reduced model with the pathway from identifiability to impact judgments constrained to 0 (Model 4) again showed that including this indirect pathway does not improve model fit, X^2^(1, *N* = 168) = 0. 6, *p* = .439. Also, as in Study 1, the differences in BICs show that Model 4 is more likely than Model 3, Pr(Model 4|ΔBIC = 4.8) = .916.

#### Path analysis: Victim number

The path analysis with impact and regret as serial mediators showed that the models with a single pathway (Models 5 & 6) are both not significantly different from a fully specified model, X^2^ (2, *N* = 168) < 2.86, *p* > .24. Although the indirect effect is marginally significant with the hypothesized order of mediators (impact → regret), *z* = –1.67, *p* = .096, and not significant for the reversed order of mediators (regret → impact), *z* = –1.36, *p* = .173, we find only weak evidence that the Model 5 is superior to Model 6, Pr (Model 5|ΔBIC = 1.5) = .679. Using non-transformed data reveals a similar pattern of results, such that the indirect effect for Model 5 was marginally significant (*p* = .097) and not significant for Model 6 (*p* = .173).

Adding the indirect pathway through mental images and sympathy (Model 7) did not result in a better fit compared to a model that constrained this pathway to 0 (Model 8), X^2^ (1, *N* = 168) = 1.69, *p* = .193. Consistent with results from Study 1, differences in BICs suggest that Model 7 is less likely than Model 8, Pr(Model 8|ΔBIC = 3.5) = .852. Moreover, we find strong support for the hypothesized Model 5 with only one indirect pathway to be superior to the model with both indirect pathways (Model 7), Pr (Model 5|ΔBIC = 1291) > .999.

## General Discussion

The current research was designed to investigate the underlying mechanisms of two prominent findings in charitable giving: identifiability and effects of victim numbers. We were particularly interested in the mediating effects of donor- and victim-focused emotions as well as mental imagery and impact judgments. Based on theoretical arguments that mental imagery has a strong link to sympathy for victims in need of help (e.g., [3, 22]) we had hypothesized that identifying donation recipients influences donation amounts through more coherent mental imagery and higher sympathy. Conversely, manipulations of victim numbers (either as varying numbers of donation recipients or as background statistics informing about the total number of lives at risk) were expected to influence donation amounts through changing the perceived impact of a donation as well as donor-focused feelings (e.g., regretting not to donate). This hypothesis extends findings that show perceived impact of a donation to be important for explaining proportion dominance in charitable giving [29] as well as overhead aversion effects [31]. While research has emphasized these different processes as underlying motivators for people’s willingness to donate (e.g., [25, 27]), it is unclear to which extent they are complimentary or contrary in explaining identifiability and victim number effects.

Our results provide evidence for both mental imagery and perceived impact to be important in the decision to donate, however these processes are triggered differently. Perceived impact of a donation seems to be related to the number of lives at risk. This finding is in line with research on the drop-in-the-bucket effect, which states that donors are not willing to contribute if they can only help a small proportion of lives at risk [5, 9, 18, 28, 34, 41–43]. The positive effects of identifying a victim, however, seem to operate more through mental images than through impact judgments.

Based on these findings, we propose a mediation model that includes both underlying processes and demonstrates when each mechanism is more probable in influencing donation decisions. Consistent with a substantial amount of research, the present approach incorporates both affective and cognitive motivations for charitable giving, but it additionally assumes that the final impetus to give is affective in nature. Study 1 showed that the effect of impact judgments on donation amounts can be explained by donor-focused feelings and that identifiability effects operate indirectly through more coherent mental images and stronger victim-focused feelings (i.e., sympathy for the victims). Study 2 extended these findings by using real donations and a different manipulation of victim numbers (i.e., background statistics). Presenting victim statistics results in lower impact judgments, which were associated with lower donor-focused feelings and lower donation amounts.

Although we generally found support for our hypotheses, the results should be evaluated with some caution. While we found evidence across both studies for the mediating role of mental imagery and sympathy (in this order) for identifiability effects, results for victim number effects were less consistent. The difference in Studies 1 and 2 might have come from the fact that we used real donations in Study 2, which would suggest that impact judgments and regret ratings are different when real money is at stake. It is also conceivable that the change in the victim number manipulations was responsible for the different findings. The background statistics in Study 2 describe a much larger humanitarian aid crisis compared to the numbers used in Study 1. It is therefore possible that the effect of victim numbers is sensitive to the numbers used in the manipulation. Research suggests that people’s willingness to help can decrease as soon as more than one life is in danger [44], and we found slightly stronger effects for victim numbers when many lives were at risk (Study 2). While the relationship between victim numbers and perceived impact undoubtedly needs further study, it is likely that boundary conditions exist such that increases in victim numbers do not always lead to lower perceived impact.

Importantly, we did find consistent evidence across studies that the effect of impact judgments on donations is mediated by donor-focused feelings (i.e., regret). Also, in Study 2 the indirect effect of victim numbers went through impact judgments and then regret. It should also be noted that, although we used similar manipulations of victim numbers as other research (e.g., [18]), in our studies we did not find strong effects of varying victim numbers on donations. This may be due to the manipulation not being strong enough, but it may also point to the possibility that other variables influence the effects of victim numbers (such as individual differences in feelings; [44]).

Victim identification by means of pictures likely also does not have universal effects on willingness to help. The pictures themselves need to facilitate a more coherent mental image and evoke sympathy in order for the proposed mediation to work. In line with prior research on the identified victim effect [12], we manipulated identifiability by showing pictures of the victim(s). This is also similar to the common practice of charities using pictures of identified victims to solicit donations. However, further research is needed to test whether the proposed explanation of mental imagery and victim-focused feelings (e.g., sympathy) also hold for other manipulations of identifiability [17]. Some authors argue that merely determining a victim (without further identification) can increase prosocial behavior even if they are not presented in a way that increases their vividness [17, 45]. Our results speak specifically to the effect of using visual imagery, however it is unclear whether merely determining a victim has the same effect.

Moreover, in the current manuscript we treat identifiability and victim number effects independently, although an argument can be made that it is easier to identify (and mentally represent) smaller groups of victims compared to larger groups [3, 46]. Indeed, some evidence exists that, next to effects of identifiability, effects of victim numbers (e.g., singularity) are also related to mental imagery. Results of Kogut and Ritov [12] can be explained by stronger mental imagery for single victims; however this is especially the case when single victims were also identified.

Another potential limitation is that we measured the constructs in this paper with single items. While single items can be successfully used to measure constructs that are sufficiently narrow [47], future studies should also assess perceived impact, mental imagery, and feelings with multiple-item scales to increase reliability. For example, multi-item measures of mental imagery could distinguish between vividness and ease of imagination as precursors of victim-focused feelings.

Given the correlational nature of the mediating variables, the current analyses cannot definitely specify a causal direction between the mediators. However, the findings in our mediation analyses are consistent with existing literature (e.g., [3, 23]) assuming that cognitive processes such as mental imagery and impact judgments influence the generation of feelings. Nevertheless, a reverse causal order is also conceivable (although theoretically unlikely) such that people experience feelings and tailor their impact judgments in a congruent way.

Finally, the mediation models that we test in this manuscript are primarily designed to explain donations towards individual (and small groups of) victims. Future research should investigate whether the same explanations also hold for donations towards charitable organizations. The presence of other victims that cannot be helped seems to reduce the willingness to donate when the recipients are individuals [48] but this may not the case when the recipient is a charitable cause or an NGO. Moreover, other research reports that under some conditions empathy mediated donations to a single victim but not to charitable organizations [49].

## Conclusions

Given the prominence of private donations for nonprofit organizations in the humanitarian aid sector, it is vital to understand the foundations of people’s decisions to donate to charity. Discovering the different psychological mechanisms and motivations in all their complexity constitutes a particular challenge for researchers. Donation decisions are influenced by both cognitive and affective processes, which can lead to non-normative and sometimes even irrational giving behavior. The mediation model presented in this paper suggests that the underlying mechanisms responsible for such non-normative donation behavior are different and specific to how victims are presented. Stating the total number of lives at risk can have a negative effect on donations (via impact judgments and regret), while identifying victims can increase donations through stronger mental imagery and sympathetic responses.

## Supporting Information

S1 Dataset Data for Study 1. (SAV)

S2 Dataset Data for Study 2. (SAV)
